# Enablers and barriers to effective diabetes self-management: A multi-national investigation

**DOI:** 10.1371/journal.pone.0217771

**Published:** 2019-06-05

**Authors:** Mary D. Adu, Usman H. Malabu, Aduli E. O. Malau-Aduli, Bunmi S. Malau-Aduli

**Affiliations:** 1 College of Medicine and Dentistry, James Cook University, Townsville, Australia; 2 College of Public Health, Medical and Veterinary Sciences, James Cook University, Townsville, Australia; University of Auckland, NEW ZEALAND

## Abstract

**Objective:**

The study aimed to identify the common gaps in skills and self-efficacy for diabetes self-management and explore other factors which serve as enablers of, and barriers to, achieving optimal diabetes self-management. The information gathered could provide health professionals with valuable insights to achieving better health outcomes with self-management education and support for diabetes patients.

**Methods:**

International online survey and telephone interviews were conducted on adults who have type 1 or type 2 diabetes. The survey inquired about their skills and self-efficacy in diabetes self-management, while the interviews assessed other enablers of, and barriers to, diabetes self-management. Surveys were analysed using descriptive and inferential statistics. Interviews were analysed using inductive thematic analysis.

**Results:**

Survey participants (N = 217) had type 1 diabetes (38.2%) or type 2 diabetes (61.8%), with a mean age of 44.56 SD 11.51 and were from 4 continents (Europe, Australia, Asia, America). Identified gaps in diabetes self-management skills included the ability to: recognize and manage the impact of stress on diabetes, exercise planning to avoid hypoglycemia and interpreting blood glucose pattern levels. Self-efficacy for healthy coping with stress and adjusting medications or food intake to reach ideal blood glucose levels were minimal. Sixteen participants were interviewed. Common enablers of diabetes self-management included: (i) the will to prevent the development of diabetes complications and (ii) the use of technological devices. Issues regarding: (i) frustration due to dynamic and chronic nature of diabetes (ii) financial constraints (iii) unrealistic expectations and (iv) work and environment-related factors limited patients’ effective self-management of diabetes.

**Conclusions:**

Educational reinforcement using technological devices such as mobile application has been highlighted as an enabler of diabetes self-management and it could be employed as an intervention to alleviate identified gaps in diabetes self-management. Furthermore, improved approaches that address financial burden, work and environment-related factors as well as diabetes distress are essential for enhancing diabetes self-management.

## Introduction

Diabetes mellitus is a major public health problem with rapidly increasing prevalence. In 2017, the global prevalence of diabetes among people aged 20–79 years was 425 million, mainly comprising type 1 or type 2 [1]. Diabetes is one of the top 10 global causes of mortality. In 2015, it was responsible for 1.6 million deaths, indicating a 60% increase in 15 years from less than 1 million in 2000 [2]. International audits have found that regimen adherence is less than optimal in both types 1 and 2 diabetes patients [3]. As a consequence, the majority of these patients are at risk of serious health complications that endanger life [1, 4] and impose great economic burden on affected individuals and the health care system [1].

Consistent engagement in diabetes self-management has been found to be correlated with the attainment of health outcomes in terms of good blood glucose control, fewer complications [1, 5], improved quality of life [6, 7] and reduction in diabetes-related death risks [8]. The term “self-management” refers to day to day activities or actions an individual must undertake to control or reduce the impact of disease on their health and wellbeing [9] in order to prevent further illness [10]. Diabetes self-management actions involve engagement in recommended behavioural activities such as healthy eating, medication adherence, being active, monitoring, reducing risks, problem-solving and healthy coping, which are all necessary for the successful management of the disease [11]. Level of adherence to diabetes self-management differs in patients, which implies that decision-making processes for self-management are influenced by various factors, which could either serve as enablers or barriers.

### Enablers of self-management

Enablers of self-management are mechanisms or factors that foster the ability of patients to undertake their recommended self-management regimen. Such factors are diverse and include effective social support with assistance and encouragement from family members [12, 13] or peers who have diabetes or close relatives familiar with its management [14]. Likewise, individual resolution to prevent or reduce the risk of developing diabetes complications helps with the determination to engage in self-management [12,15]. Studies have also noted positive decision making about diabetes self-management as a result of effective health care provider-patient communication [16], characterized by trust, respect and shared decision-making in planning health goals [17, 18]. In addition, patient support with the use of health technological interventions such as mobile phone applications [19] and self-management education [20, 21], facilitate efective diabetes management. Individual factors, particularly higher educational level [22, 23] and gender [24], also contribute to patients’ ability to care for their diabetes.

More importantly, adequate self-management skills [25] and self-efficacy (confidence) [26] to perform these skills are major enabling factors for engagement in diabetes self-management. This is because skills and self-efficacy operate in tandem to foster full engagement with self-management. Self-management skills result from knowledge about the disease [25], and understanding the interrelationships between different self-management activities and their impact on health outcomes [27]. On the other hand, self-efficacy refers to ‘‘one’s belief in his/her own innate ability to perform specific tasks required to reach a desired goal” [28]. Unless people believe they can produce desired effects by their action, they have little incentive to act [29], regardless of other enabling factors which may be available to them. In diabetes management, patients’ level of self-efficacy is influenced by their level of skills for self-management. Hence, patients with adequate skills and efficacy have more likelihood to adhere to prescribed behavioural regimen necessary to attain optimal health [25, 30–32]. Acquiring diabetes self-management skills and efficacy is an ongoing learning process [20, 25]. While some skills and efficacy are easily acquired, others are often difficult to attain. Further research is therefore needed to adequately identify gaps in diabetes patients’ skills set and self-efficacy levels for self-management of their health issues. Information on identified gaps will guide health care providers in their development of educational support programs that foster self-management among diabetes patients.

### Barriers to self-management

Non-adherence to recommended diabetes self-management regimen is influenced by barriers encountered by patients. These barriers make managing the disease more difficult. Only few studies have examined patients’ perceived barriers to general diabetes self-management from a global perspective. An international study identified diabetes related distress as a major factor responsible for poor adherence to self-management in patients [3]. Local studies reported that difficulty in making lifestyle changes [33] and inadequate health care system communication interface [34] were related to poor diabetes self-management. In addition, financial constraints resulted in patients’ inability to access diabetes clinical supplies and eat in line with appropriate dietary recommendations [35–37]. Other studies have examined barriers to some specific areas of diabetes self-management. Nagelkerk et al., [16] and Ghimire [38] reported that patients’ lack of knowledge of a specific diet plan and perceived belief in social unacceptability of healthy behaviours hindered healthy eating and participation in physical exercise. Furthermore, depressive symptoms and personal belief about medication were observed to be associated with lower adherence to diabetes medications [39].

The empirical and conceptual research findings mentioned above are not exhaustive because only a few have an international focus [3]. Additionally, the studies are mostly focused on barriers to self-management in patients with type 2 diabetes only [33–36, 38, 40], older populations [35], those from low income background without indicating the type of diabetes the respondents had [41], or few areas of diabetes self-management [38–40]. The above limitations in previous studies emphasize the need for further and detailed exploration of factors serving as barriers to self-management in both types 1 and 2 diabetes patients. This will provide strategies that adequately address such challenges and foster better adherence to self-management for better health outcomes in both patient groups.

### Study aims

There is diversity in the level of self-management between patients. The ability to self-manage diabetes is influenced by various factors that can either serve as enablers or barriers. However, to the best our knowledge, global perspectives on the crucial enablers of self-management in terms of skills and self-efficacy, among types 1 and 2 diabetes patients is relatively scarce. Likewise, studies on other enablers and potential barriers to general self-management as perceived by these patient groups is scanty in the published literature. There is special interest in elucidating this information from an international perspective because issues encountered in self-management by both patient groups are likely to include common experiences and challenges. Identifying these commonalities could provide health professionals with an in-depth understanding of patients’ experiences and help guide the development and enhancement of intervention strategies to improve patients’ self-management of diabetes. Therefore, this study aimed to: i) identify the common gaps in skills and self-efficacy for self-management among individuals with type 1 or type 2 diabetes; ii) examine factors associated with self-management skills and self-efficacy; iii) explore other factors which serve as enablers of, and barriers to, achieving optimum diabetes self-management.

## Methods

### Recruitment procedure

A maximum variation purposive sampling technique was employed in recruiting participants aged ≥ 18 years who had type 1 or type 2 diabetes. Participants were recruited globally using diverse recruitment strategies. The aim of this sampling method was to obtain a mix of participants with diverse experiences and identify common patterns that cut across the population sample with regards to the subject of interest [42]. Officially approved advertisement for the study was placed on various health organizations’ websites. These websites included Diabetes UK and Diabetes Australia. In addition, the advertisement was placed in local digital newspapers, Twitter and Facebook pages focusing on diabetes support. Data collection was conducted between November 2017 and June 2018. There was no limit to sample size in order to capture the maximum number of people with type 1 or type 2 diabetes. The study requested participants’ socio demographic characteristics of age, gender, educational level and geographic location. Details of the recruitment strategy and participants’ characteristics have been fully described in our previous publication [43].

### Study design

A sequential mixed methods approach was used; comprising quantitative and qualitative data collection methods [44]. The quantitative phase of the study involved a cross sectional survey and data analysis. This was followed by qualitative telephone interviews of a subsample of the participants in order to provide a more complete and comprehensive understanding of the results which were integrated into the data interpretative phase [44]. Quantitative data were obtained through an online survey that focused on assessing participants’ self-reported skills and self-efficacy (confidence) as part of the factors that enable diabetes self-management. Qualitative data were collected through individual telephone interviews which further explored additional factors that serve as enablers and barriers to diabetes self-management.

#### Quantitative measures–survey

The survey questions were divided into two parts. First, the following health characteristics which were likely to influence skills and self-efficacy for diabetes management were assessed: type of diabetes, duration of diagnosis and whether participants had recently received (within the previous 12 months) diabetes self-management education (DSME) from a member of their health care team.

Second, novel LMC Skills, Confidence and Preparedness Index (SCPI) tool was used to assess skills and self-efficacy in core behaviours central to diabetes self-management such as healthy eating, blood glucose monitoring, being active, healthy coping, medication adherence, problem solving and reducing risk [11, 45]. The SCPI tool had been previously validated, where its construct validity for different ages, ethnicity, gender and level of education was established [32]. Additionally, the validity of the tool for use in different settings is established by the fact that, as a new tool, the questions reflect the current recommended self-management regimen for diabetes patients, and this has not been fully explored by previous tools [45]. It has excellent readability and reliability. Permission was obtained to use the tool. The SCPI tool consists of three subscales: skills, confidence and preparedness. The skills subscale was used to assess perceived ability to perform the self-management activities mentioned above. The confidence subscale was used to assess self-efficacy in being able to perform the skills. The preparedness scale was not used in this study because this subscale assesses the readiness of patients to implement behavioural changes following an educational session; which was not applicable in the present study.

The skills and confidence domains consist of nine (9) and eight (8) items respectively. Two of these items focus on skills and confidence to use insulin. These skills were adapted to accommodate participants who have type 2 diabetes but do not use insulin/other medications as part of their treatment regimen. All items were rated using a visual analogue scale, with scores between 1 and 10. Each of the items in the domains produced its own score out of 10. The total score was the mean score in each of the subscales, where higher scores denoted better skills and confidence. The scoring process is not affected by demographic factors such as age, gender, level of education or ethnicity [45], hence, its’ applicability for use in study populations with diverse social and health characteristics. The instrument was administered in English Language.

#### Qualitative measures–phone interviews

Through online survey, all participants were invited to an individual telephone interview session. They were requested to indicate interest by providing their best contact number and availability. A single independent resource person (male) who is an experienced researcher in qualitative studies conducted all interviews. The interviewer was trained on the aims of the study and the interview guide by the first author of this study (MDA). The guide was then pilot tested between the interviewer and MDA before actual use. Additionally, MDA was present in the first three interviews to ensure appropriateness of data collection. While the interviews were used to reflect on the interview guide, no changes were made to the guide afterwards. There was no interaction or previous relationship between MDA and the participants. The interviewer was located in a private office at James Cook University, Townsville, Australia. Prior to the commencement of the interview, each respondent was asked if they were located in a comfortable place for an interview, and were briefly presented with the general idea of the study and key diabetes self-management activities. The interviewer did not have prior relationship with the participants. Each Interview was audio recorded and lasted between 7 and 20 minutes in duration. Data saturation was achieved through recurring explicit ideas [46] after completing the 14^th^ interview. However, the interview was conducted for the remaining two participants who had indicated interest in order to ensure that no main idea was unintentionally discarded. Repeat interviews were not required and due to the remoteness of the study participants, there was no post interview debriefing. The semi-structured interview guide was developed by the research team. Topics covered in the interview included open ended questions and probes to facilitate discussion (See S1 Appendix for details of the interview questions).

### Ethics and consent

The study procedures (registration number: H7087) were approved by James Cook University’s Human Research Ethics Committee. The protocol contained detailed information on the ethical obligations of researchers toward participants engaging in online research activities. Essentially, these obligations included confidentiality, anonymity, scientific value, maximising benefits, minimizing harms, and informed consent [47]. All these obligations were strictly adhered to during the research process. Furthermore, as part of the application process for advertisement of the study on the website of health organisations, the ethics approval document was made available to the appropriate and designated officials of these organisations. All prospective study participants were provided with the study information along with the privacy policy prior to the survey. Therefore, participants were informed about the use of their answers for analysis under anonymity. Informed consent was implied by submission of the online survey, while all telephone interviewees provided verbal consent.

### Data analyses

SPSS (Version 23) was used for quantitative data analysis. Cronbach’s alpha of the subscales of measures used in this study were acceptable (.92 and .91 for skills and confidence scales respectively). Participants’ demographics and health variables were presented using descriptive statistics. Items in the skills and self-efficacy domains were reported as means and standard deviations (SD). For the purpose of explaining and discussing the results, scores were graded as high (≥ 7), moderate (4–6) or poor (≤ 3). Mean scores were calculated for demographic and health variable subgroups. Bivariate analyses were performed using Independent sample t-test and Analysis of Variance (ANOVA) to test the relationship between participants’ subgroups and level of skills and confidence. Specifically, t-test was used for variables with two categories (i.e. type of diabetes, received DSME or not, gender) while ANOVA was used for variables with three or more categories (i.e. educational status, duration of diagnosis, geographic location, age range). Effect sizes were calculated using Eta squared values to show the magnitude of difference in mean scores between categories within each variable. Pearson correlation coefficients were used to estimate the strength of association between skills and self-efficacy scores. Additionally, multiple regression analysis was used to estimate the contributions of the different independent variables to participants’ reported skills levels. Significant variables in the bivariate analysis were included in the regression. In all statistical analysis, values were considered statistically significant at *p*< 0.05 (two tailed).

For qualitative data analysis, audio recordings were transcribed verbatim by an independent professional transcriber and reviewed by the first author (MDA) for accuracy. The transcripts were uploaded into a qualitative data analysis software (QSR NVivo 11). Emerging themes were identified using in-depth inductive thematic analysis [48] undertaken in six steps: (i) re-reading of data line by line to ensure familiarization (ii) identification of patterns within data and organization into codes (iii) grouping of initial codes through constant comparison to identify emerging themes (iv) grouping and review of identified themes into general themes (v) refining themes and (vi) selection of representative quotes to support themes [48]. The first coding and generation of themes was done by MDA. In order to enhance result credibility and validity, raw data transcripts, coded data and themes were independently reviewed by the last-named author (BMA). Data were cross-checked in a consensus meeting and there was 90% degree of congruence between both authors’ coding, themes and classifications. Discrepancies were resolved through discussion and mutual agreement. Both MDA and BMA have experience in qualitative research methods. The remaining two researchers (UMA and AEOMA) checked the quotes and themes to ensure consistency. Key themes were reported along with relevant quotes affixed with an assigned number code and the type of diabetes the respondent has (for instance P3, T2D). The final manuscript was subjected to COREQ checklist for consolidated criteria for reporting qualitative research (See S1 Checklist) [49].

## Results

### Socio-demographic and health characteristics

A total of 217 complete responses to the online survey was received. Respondents were located in four geographic regions; namely, Europe (35%), Australia (34.6%), Asia (29.5%) and America (0.9%). The mean age of respondents was 44.65 ± 14.0 years (range 18–76 years) and 56.7% of them were females. More than half of the respondents had type 2 diabetes (61.8%) and had received DSME in the previous 12 months prior to the study (64.1%). About half of them were diagnosed in the last 5 years (52.5%) while 20.3% were diagnosed 6–10 years ago and the remaining 27.2% over 10 years. Over half of the respondents (56.2%) reported having a minimum of bachelor’s degree, 20.3% completed high school, while 18.9% completed technical college and 4.6% attained other forms of education.

A total of 31 respondents (14.3%) expressed interest to participate in the telephone interview. However, about half of them declined at time of interview or never responded to phone calls, leaving a final respondent number of 16 individuals who were interviewed. The participants were mostly males; 56.2% (9/16), had type 1 diabetes; 62.5% (10/16) and lived in Australia; 87.5% (14/16), with age ranging from 26 to 61 years [mean age of 44.56 (SD 11.51)].

### Diabetes self-management skills and self-efficacy (confidence)

Table 1 shows the mean scores for each of the items across the skills and self-efficacy domains. Scores were highest in the skills for knowing the appropriate time to check blood glucose levels in order to reflect either the impact of meals consumed ($\bar{x}$ = 7.81 ± 2.33) or medications/physical activities ($\bar{x}$ = 7.47 ± 2.37). In addition, participants possessed a high ability to recognize the effect of missed physical activity or excess carbohydrate consumption on their health and knew the corrective steps to take ($\bar{x}$ = 7.35 ± 2.35). The lowest scores were in the areas of skills for: identifying and managing the impact of stress on diabetes ($\bar{x}$ = 6.88 ± 2.43), exercise planning to avoid hypoglycemia ($\bar{x}$ = 6.88 ± 2.48), and interpreting blood glucose patterns ($\bar{x}$ = 6.84 ± 2.58).

**Table 1:** Participants skills and self-efficacy (confidence) ratings to perform diabetes self-management.

Skills	Mean	SD
I am able to portion out and choose foods that have the minimal balance between carbohydrates, proteins and vegetables to keep my blood sugar on target	7.23	1.97
I know how my diabetes insulin and medication works in my body and at what time of the day I should check my blood sugar (BS) to make sure my dose is correct (*For T2D*^*a*^ *not controlling with insulin and medication*: I know how my diet and physical activities impact my BS and at what time of the day to check my BS to make sure they are on target)	7.47	2.37
If I eat too much carbohydrate, or do not engage in my regular physical exercise, I know how my body will react and the steps to take to get it back on track	7.35	2.35
When I am planning to exercise, I know what changes I need to make to avoid low blood sugar before, during and after exercise	6.88	2.48
I know when to check my blood sugar if I wanted to see how my body reacted to a meal	7.81	2.33
When I am sick, I know what to do differently with my medications, fluids intake, food intake, blood sugar testing and when to go to the hospital	6.91	2.67
I know how to identify stress in my life and how it can impact my diabetes management and overall health	6.88	2.43
When I look at my blood sugar in my meter or in my log book in a given week, I could explain to my diabetes educator or doctor what my blood sugar pattern is	6.84	2.58
I know what the ABCs (HbA1c^b^, Blood Pressure and Cholesterol) of diabetes are, what my targets are and how they impact my diabetes	7.00	2.54
***Average score on skills***	7.15	1.97
**Self-Efficacy**		
I feel confident that I can plan meals and snacks effectively in a way that it will not raise my blood sugar unnecessarily above my targets	7.22	2.06
I am confident that I can implement stress management techniques in my lifestyles	6.72	2.28
I am confident that at the next time I am eating out in my home, I will be able to plan and select the foods that best keep my blood sugar under control	7.06	2.34
I am confident that I can plan ahead for what to do and how to react either before, during or after exercise to avoid a low blood sugar	6.92	2.4
I am confident that I can choose a healthy physical activity for myself and include it in my schedule	7.16	2.26
I am confident that I can adjust my insulin or medication doses on my own, to reach the target blood sugar levels *(For T2D*^*a*^ *not controlling with insulin and medication*: I am confident that I can adjust my meals and levels of physical activities on my own to reach the target blood sugar levels)	6.87	2.62
I am confident that I can commit to preventing and monitoring my diabetes complications such as seeing my eyes doctor at least once in a year and checking my feet on daily basis	8.08	1.85
I am confident that I can use my blood sugar results to make changes to my diet and/or insulin to help keep my blood sugar in target	7.00	2.54
***Average score on confidence***	7.17	1.81

^a^T2D: Type 2 diabetes mellitus

^b^HbAIC: Glycosylated hemoglobin

In relation to participants’ self-efficacy levels, the highest scores were in confidence to reduce risk by preventing and monitoring diabetes complications ($\bar{x}=$ 8.08 ± 1.85), and using blood glucose results to plan for meal intake ($\bar{x}=$ 7.22 ± 2.06). Participants scored lowest in their confidence for healthy coping with stress ($\bar{x}=$ 6.72 ± 2.28) and adjusting medications or food intake to reach targeted blood glucose levels ($\bar{x}=$ 6.87 ± 2.62).

There was a strong positive correlation between the scores in the two domains, *r* = .906, *p<0*.001, where higher levels of perceived skills were associated with higher levels of perceived self-efficacy. Coefficient of determination (R^2^) indicates that level of skills explained 82% of the variation in respondents’ scores on self-efficacy.

#### Relationship between participants’ characteristics and levels of skills and self-efficacy

Table 2 shows the relationship between demographic and health characteristics and the levels of skills and self-efficacy for diabetes management in participants. All demographic characteristics except geographic location, gender and age, were significantly associated with perceived skills and self-efficacy.

**Table 2:** Summary of t-test or ANOVA and post hoc results on mean scores by participants’ characteristics.

	Skills			Self-Efficacy		
Variables	mean ± SD	*F* or *t* statistics, *p*-value	Effect size	mean ± SD	*F* or *t* statistics, *p*-value	Effect size
**Type of Diabetes**		t(215) = 17.41,*p*<0.001	0.123		t(215) = 5.46, *p* = 0.02	0.051
Type 1	7.95 ± 1.35			7.66 ± 1.47		
Type 2	6.66 ± 2.12			6.87 ± 1.93		
**Duration of diagnosis (years)**		*F* (4) = 5.59, *p*<0.001	0.095		*F* (4) = 3.23, *p* = 0.013	0.057
<1	6.28 ± 1.82			6.50 ± 1.68		
1–5	6.98 ± 2.08			7.12 ± 1.86		
6–10	6.97 ± 2.14			6.96 ± 2.15		
10–15	7.00 ± 1.58			7.20 ± 1.27		
>15	8.28 ± 1.22			7.95 ± 1.30		
**Received DSME**^**a**^ **in the previous 12 months**		t(215) = 1.89,*p* = 0.045	0.018		t(215) = 1.48, *p* = 0.141	0.01
Yes	7.35 ± 1.77			7.31 ± 1.66		
No	6.79 ± 2.23			6.93 ± 2.03		
**Educational status**		*F* (4) = 7.87, *p*<0.001	0.132		*F* (4) = 6.77, *p*<0.001	0.113
High School	6.13 ± 2.21			6.42 ± 1.98		
Technical College	6.53 ± 2.25			6.47 ± 2.11		
Bachelor Degree	7.47 ± 1.65			7.48 ± 1.39		
PG degree^b^	7.76 ± 1.53			7.71 ± 1.55		
Others^c^	8.40 ± 1.12			8.18 ± 1.46		
**Gender**		t(215) = -1.18,*p* = 0.238	0.006		t(215) = -0.43, *p* = 0.665	0.001
Male	6.97 ± 1.93			7.11 ± 1.76		
Female	7.28 ± 1.99			7.22 ± 1.85		
**Geographic location**		*F* (3) = 3.14, *p* = 0.124	0.005		*F* (3) = 3.22, *p* = 0.145	0.004
Australia	7.11 ± 2.01			6.96 ± 1.87		
Europe	6.73 ± 2.40			6.91 ± 1.94		
America	6.99 ± 2.11			6.77 ± 2.10		
Asia	7.16 ± 2.10			6.76 ± 2.13		
**Age**		F (4) = 1.46, *p* = 0.215	0.027		F (4) = 1.48, *p* = 0.211	0.027
18–29	6.95 ± 1.78			6.88 ± 1.80		
30–39	7.60 ± 1.52			7.52 ± 1.58		
40–49	7.06 ± 2.16			7.01 ± 2.02		
50–59	6.74 ± 1.98			6.81 ± 1.64		
60–69	7.12 ± 2.42			7.39 ± 2.05		

^a^DMSE: Diabetes Self-Management Education

^b^Post Graduate

^c^Others: Professional qualifications, graduate diploma

Participants who had type 1 diabetes had higher levels of skills compared to those with type 2 diabetes, *t* (215) = 17.41, *p*< 0.001, eta squared = 0.123. Additionally, receiving DSME within the past 12 months prior to participating in the study had a moderate but significant association with level of skills, *t* (215) = 2.01, *p* = .045, eta squared = 0.018. There was a significant difference in duration of diabetes diagnosis, *F* (4, 215) = 5.59, *p*<0.001, eta squared = 0.095. Skill scores were significantly higher in the >15 years (*M* = 8.28, *SD* = 1.22) when compared to <1 year (*M* = 6.28, *SD* = 1.82), 1–5 years (*M* = 6.98, *SD* = 2.08) and 6–10 years (*M* = 6.97, *SD* = 2.14) of diabetes diagnosis. There was no significant difference for those with 10–15 years of diagnosis (*M* = 7.00, *SD =* 1.58). In addition, level of educational qualification significantly influenced the level of skills, *F* (4, 215) = 7.87, *p*<0.001, eta squared = 0.132. Skill scores were significantly higher among postgraduate degree holders (*M* = 7.76, *SD* = 1.12) in comparison to high school (*M* = 6.13, *SD* = 2.21) and technical school (*M* = 6.43, *SD* = 2.25) certificate holders. No significant difference was observed when compared to those with bachelor’s degree (*M* = 7.76, *SD* = 1.53).

For self-efficacy (confidence), type 1 diabetes participants had higher confidence levels compared to their type 2 counterparts, *t* (215) = 5.46, *p* = 0.02, eta squared = 0.051. Furthermore, confidence score was significantly associated with duration of diagnosis, *F* (4, 215) = 3.23, *p* = 0.013, eta squared = 0.057. Confidence was significantly higher in the >15 years (*M* = 7.95, *SD* = 1.30) when compared to <1 year (*M* = 6.50, *SD* = 1.68) only. Furthermore, level of educational qualification significantly influenced confidence level, *F* (4, 215) = 6.77, *p* <0.001, eta squared = 0.11. Participants with postgraduate degree had significantly higher confidence (*M* = 7.71, *SD* = 1.55) in comparison to those with high school (*M* = 6.42, *SD* = 1.98) and technical school (*M* = 6.47, *SD* = 2.11) certificates. No significant difference was observed for those with bachelor’s degree (*M* = 7.48, *SD* = 1.39).

Multiple regression analysis identified the simultaneous contributions of duration of diagnosis, type of diabetes, educational qualification and receiving DSME within 12 months prior to the study on participants’ level of skills. These variables predicted 22% of the variation in level of skills *F* (2, 216) = 14.815, *p*<0.001, *R*^*2*^ = .218. All variables, except receiving DSME, were statistical significant at *p* < .05.

### Other enablers of self-management

Two major themes were identified as factors which could facilitate diabetes self-management. These were patients’ determination to prevent the development of complications and the use of health technological devices or software.

#### 1. Determination to prevent diabetes complications

The decision to regularly engage in self-management was fostered by participants’ resolution to prevent the development of diabetes complications. Participants ensured that they engaged in the necessary lifestyle behavioural activities due to their determination to maintain better quality of life and thereby avoid what was observed in their peers who had already developed some form of diabetes complications:

*‘‘I see a lot of other people who already have diabetes talking about their diabetes on social media*. *Looking at others who are worse off than me and the problems they struggle with*, *I guess is keeping me in check saying*, *hell no*, *I’m not going down that path”*. [P6, T2D]

Furthermore, the determination to prevent diabetes complication was expressed by refusal to purchase certain foods which participants believed could increase the risk of progressing type 2 diabetes management into requiring the use of insulin injection:

*‘‘It’s just the fact that I don’t want to get to the stage of having injections many times a day…*. *I have to remind myself of that always*. *I’m quite happy to walk past some chocolate…*.knowing fully well that whilst I might enjoy a ** (name of a chocolate brand), *…*. *then I get an injection at the end*, *which I don’t want*, *which mean I will leave (name of a chocolate brand) alone”*. [P3, T2D]

Respondents acknowledged that having good knowledge and problem solving skills in diabetes has proven useful to aid their self-management. Awareness of how foods impact their health was reported as highly essential:

*‘‘I think having knowledge of the foods and the type of foods and diet and portion sizes are very important*. *Also*, *I found understanding what hypo or hyper*, *and understanding how my body reacts and how I can resolve that has been very useful in managing my diabetes*.*”* [P15, T1D]

#### 2. Use of health technological software and devices

Participants mentioned the use of mobile technological devices specifically, smart phone application (apps), insulin pump and continuous glucose monitors (CGM) as supporting tools which have enhanced their self-management.

2.1 APPS: Some of the participants use smart phone apps to record their blood glucose data. They noted that having access to such previously stored data on their phones gave them insight into the best self-management strategy which had assisted in adequate glycemic control:

*‘‘I have been diagnosed for a long time and back in the days I used to write it on a note book*. *But in these days*, *I record it using a smart phone app*, *which allows me to search*. *So it allows me to access the data quickly and make a sort of best guess for now based on what happened in the past*. *If it’s not working*, *as it has done recently*, *I can go back to strategies that I might have been using years ago*, *that seems to work then “*. [P14, T1D]

Reminder feature in apps were found useful to give alert for recurring tasks such as taking medications thereby improving medication taking behaviour especially during busy schedules:

*‘‘I’m only kind of new to this (newly diagnosed)*, *so I am actually looking for ways to remind myself of the tablets (medication) am meant to be taking*. *When I get really busy I forgot*‥*so my app pings at me a certain time of the day…just to kind of prompt me”* [P3, T2D]

Also, motivations and encouragements were received through the use of app especially in the event of unstable blood glucose control. Participants stated that whenever their blood glucose level fluctuated and differed from the prescribed limits despite all efforts to stabilize it, looking at good data previously stored in apps provided an assurance that their blood glucose levels will not always be unstable:

*‘‘Sometimes it is simple as realizing it’s not all terrible*. *Being able to flip back on my smart phone*. *If you’ve had a rough four or five days*, *it can feel like it’s a long time since you’ve seen numbers that felt like relatively stable or in range*. *You can get disheartened but if you can just check back you and see*, *actually no*, *it’s fine because two weeks ago it was all right*, *so I’ll be able to get back to that again*. *So having access to that sort of information storage allows me to be a little bit more relaxed when inevitable things start to wobble and go adrift again”*. [P14, T1D]

2.2 INSULIN PUMP AND CGM: Participants with type 1 diabetes reported the use of insulin pump or continuous glucose monitor (CGM) as external aids which made it easier for them to manage and effectively monitor their health. In this regard, one participant stated that:

‘‘With the insulin pump, I find it easier to manage. Also, I’ve got the CGM and I can see what my sugar is on the screen all the time….you know that changed my life”. [P1, T1D]

Participants also indicated that use of insulin pump provided additional support and relief from pains experienced while using needles:

‘‘*I’m quite a thin bloke…… and have no body fat so inserting needles really hurt*. *My insulin pump definitely helps*. *So the best way I’ve managed my diabetes is through the insulin pump”*. [P12, T1D]

### Barriers to self-management

In spite of the factors that foster effective self-management of diabetes, the key themes that emerged from the interview indicated that people with diabetes encountered diverse challenges in performing their self-management due to the: i) dynamic and chronic nature of diabetes; ii) financial constraints iii) work and environment related factors; and (iv) unrealistic expectations

#### Theme 1: Dynamic and chronic nature of diabetes

The most common complaint reported by participants was the dynamic and chronic nature of diabetes and how these attributes make diabetes self-management require multiple needs. Participants felt there were many reasons including environmental conditions, which may demand an adjustment in their self-management even within short time periods. They believed the constant requirement to modify needs of the condition denoted certain things they were not doing right in their self-management and they always had to put in great effort to meet up with their health requirements:

*‘‘Because I live with type 1 diabetes I have to do a complete insulin replacement*, *which involves balancing for activity*, *ambient temperature*, *stress levels*, *insulin sensitivity of my body*. *It could be so much easier if you could just work out what your insulin to carb sensitivity portion is*, *work out how to behave around exercise*, *work out correction factors and that would be all*. *But no*, *my experience is that that’s it for a week and then your basal requirement would have changed*. *Then the weather get warmer*, *you may need to re-evaluate your insulin sensitivity and carb ratio*. *So it’s just- you are never getting it right and you’re just always constantly trying to play catch up”*. [P14 T1D]

Likewise, the effects of self-management on diabetes outcome was referred to as a system which could not be automatically controlled. Participants described how similar behavioral activity such as eating the same diet over time could impact their health differently.

*‘‘It is a dynamic disease*. *I mean what works today doesn’t work tomorrow*. *You can eat something today and you can be okay*, *eat something tomorrow and it can be completely different*. *So you can never just put it on a cruise control and away you go”*. [P2, T1D]

The weariness about the never-ending need for self-management because diabetes is a lifetime disease was expressed:

*‘‘The biggest thing that fazes me is just the fact that it’s something that you have to do 24 hours a day*, *seven days a week and nothing ever going to change that”*. [P4, T1D]

Participants were sometimes unwilling to undertake their self-management because they felt it is not a permanent cure for the disease, diabetes is chronic, so what is the point?:

*‘‘*‥*Probably my mind frame*, *in just getting yourself down to the fact that it’s never going to*‥*I’m always going to have it*. *So you sort of question what’s the point (of management)*? *It’s hard to comprehend”*. [P11, T1D]

The presence of other diabetes related complications or health problems such as neuropathy and depression in some participants limited their ability to actively engage in behavioral activities especially physical exercise or healthy eating:

‘*‘Physical exercise is difficult…Yeah*, *I have peripheral neuropathy of the leg*, *a collapse in the foot and yeah*, *problems with the other foot”*. [P10, T2D]

*‘‘Nutrition is something that is hard to keep on top of*. *I suffer from a major depressive disorder*, *so I have a lot more trouble following my optimum diet”*. [P7, T1D]

#### Theme 2: Financial burden

The difficulty in meeting the financial cost for some diabetes medical tests and other treatment requirements was also identified as a barrier. Participants voiced out the financial burden they experienced by citing the need to pay for some clinical tests and diabetes supplies which are not covered by their health insurance such as the glycosylated hemoglobin (HbA1c) test and continuous glucose monitor. They expressed the desire to receive more support from the government:

*‘‘I manage my diabetes fairly closely and I pay for HbA1c*, *you know …the financial cost is quite large*. *In Australia*, *our health system’s pretty good but you still have to pay for a lot of equipment which the government doesn’t seem to agree necessarily*. *Continuous Glucose Monitor should be government funded for over 21s for Christ sake”*. [P2, T1D]

Another participant based in the United Kingdom (UK) stated:

*‘‘I don’t have unimpeded access to Continuous Glucose Monitor (CGM)*. *I mean*‥*the situation of health care in UK is that it’s (CGM) not often funded by National Health Service (NHS) apart from people that are in quite profound need*. *I don’t get that assistance*…*So that’s a challenge and access issue”*. [P14, T1D]

#### Theme 3: Work and environment-related conditions

3.1: Occupation: Job requirements especially those involving a lot of travelling serves as deterrent to maintaining a healthy diet. Participants stated that the inability to get healthy choices of foods in most restaurants or public places when unavoidably required to eat out due to travelling long distances to fulfill their job requirements:

*‘‘My work requires a lot of travelling*. *If you are actually going to eat something that is actually not good and could put you in the circumstance where you know… Like I had a 16 hour travelling the other day and everywhere I turned*, *I couldn’t touch any of it*. *I had some but I had to acknowledge that it was not what I really needed to eat”* [P3, T2D]

Work related stress was also reported as a hindrance to attaining optimal blood sugar levels:

*‘‘With me personally*, *it’s stress*. *I’m an electrician*, *and I’m full time employed*, *so stress gets me*. *When I get stressed*, *my blood sugar level goes downhill”* [P13, T1D]

3.2: Weather condition: Participants find it difficult to engage in physical exercise in hot weather conditions:

*‘‘*‥*Exercise is something I have trouble getting around to doing*. *Like during the summer*, *the heat hits me big time*. *So I’m loving the cooler weather we’re starting to have because I can start to work a bit more*, *but during the heat*, *I cannot do it”*. [P2, T1D]

#### Theme 4: Unrealistic demands

Unrealistic expectations and advice about self-management from family or friends especially those not diagnosed with diabetes could be a hindrance to effective care. Participants’ found such wrong advice irritating as evident in the following comment:

*‘‘You know I don’t think a lot of non-diabetic actually get to know how much it can take to actually manage a high or a low (Blood sugar) potentially*. *You know*, *you get comments from people that you’re low and they know you are diabetic saying*, *oh*, *should you be eating that*? *Well*, *I’m going to say this nicely*, *you want me to die now or not or to go into coma*? *Because I need to eat this*. *They go oh*, *you didn’t need to say it like that*. *You go well*, *stop asking a stupid question that you don’t know anything about”*. [P4, T1D]

Additionally, discrepancy between patients and their health professionals’ (HP) perception of care could be a barrier to self-management. Participants felt that some recommendations from HPs were contrary to their opinions on what their diabetes self-management should entail:

‘*‘My doctor doesn’t feel I need to be using a glucose meter to monitor my sugar levels and the diabetes educator doesn’t think I need to be on any sort of diet*, *even though I’ve had increases in diabetes medications”*. [P10, T2D]

## Discussion

To the best of our knowledge, this is the first mixed methods study that has investigated enablers and barriers to general self-management among a multinational audience of people who have type 1 or type 2 diabetes. Most importantly, our findings emphasise the consequential impact of currency of exposure to DSME (within the previous 12 months), duration of diagnosis, level of educational qualification and use of technological devices on self-management skills and self-efficacy, regardless of geographical location or ethnicity. This implies that provision of ongoing self-management education/support through the use of mobile phones may help address the various difficulties (including time/financial constraint, diabetes distress, and limited access to care providers) encountered by patients and foster adherence to recommended self-management activities, which are necessary to prevent the risk of developing diabetes complications. Furthermore, this study presents an in-depth understanding of the experiences of diabetic patients and provides useful insights to health professionals and researchers on how to improve the frequency and quality of self-management support provided to diabetic patients to achieve better health outcomes.

### Skills and self-efficacy for diabetes self-management

The overall skills score was found to be high and many participants reported good level of ability for self-management. This is specifically in the area of accurate monitoring to assess the impact of diet, medication or physical activities on blood glucose levels. Similar findings were observed in a previous study [25]. Accurate monitoring of blood glucose in relation to foods consumed and physical activities are important because they predict good outcomes in diabetes management [50].

Although the participants in this study scored high in their ability to monitor blood glucose, their capacity to interpret their blood glucose patterns over time was only moderate. Self-monitoring of blood glucose is important to assess glycemic pattern, hence accurate interpretation of these patterns is highly important to ensure effective management of glycaemia related problems encountered in diabetes management [51]. More emphasis should be laid on glucose pattern management during diabetes self-management educational sessions in order to expatiate patients’ skills on effective monitoring and interpretation of blood glucose data and the resulting health implications.

Participants in this study possessed lower skills related to planning for physical exercise in order to avoid hypoglycemia and adjusting medication to reach targeted blood glucose levels. This result corroborates previous findings [52]. The ability to manage and make appropriate adjustment to multiple regimens often determines success with other core areas of diabetes self-management and glycemic control [51]. For instance, studies have reported that due to the fear of hypoglycemia, patients have resorted to unhealthy behaviours (such as reducing or eliminating medication dose, inappropriate food choices and /or avoiding physical activities) that increase glucose levels [53]. Diabetic patients have an increased risk of developing hypoglycemia particularly when treated with insulin or insulin secretagogues [53]. Hence, they should be provided with regular refresher courses and continuous training on blood glucose levels awareness and strategies to balance exercise which could promote glycemic control and adherence to self-management.

Healthy coping strategies to identify and manage the impact of stress on diabetes management may be a difficult aspect of diabetes care because the participants in this study scored lowest in this area for both the skills and self-efficacy domains. All forms of stress either physical or mental, negatively impact blood glucose levels in those with diabetes [54] and it is a potential obstacle to attaining effective self-management and optimal health outcomes [55]. Patients’ understanding of dimensions of diabetes related stress is a clinically important factor and forms of stress that are potentially modifiable should be prioritized to guide clinical and educational interventions. This can include regular educational information on the impact of stress on health of diabetes patients and suggestions to reduce it.

Contrary to the findings of a previous study [56] that reported people with type 1 diabetes as having poorer self-management; our study participants who had type 1 diabetes scored higher than those with type 2 diabetes in skills and self-efficacy to care for their diabetes. Additionally, there was a significant positive relationship between the duration of diabetes and both skills and confidence for self-management. Patients with type 1 diabetes are typically diagnosed at an early age that may correspond to longer duration of diabetes. This pattern might have afforded them prolonged and regular exposure to health education, which is a significant predictor of successful diabetes self-management [20].

Overall, the strong correlation between the level of skills and self-efficacy found in this study strengthens the body of evidence supporting this link [32]. This pattern may be related to high level of education among most of the study respondents as also observed in a previous study [57]. Patients who possess higher skills usually have higher perceived level of efficacy and are most likely to actually engage in their self-management [25, 32]. Building patients’ skills and confidence in their ability to self-manage diabetes is therefore imperative. Regular encouragement which could either be provided verbally or through other means of contact (e.g text messages through phones or emails) could be beneficial to patients [58]. While for those with limited educational backgrounds, the use of clear and simple communication styles when providing diabetes education to them will be essential to foster their skills and confidence [57].

### Other enablers of self-management

Based on the results of the interviews, the most commonly perceived factor that fostered regular self-management was the will to prevent the development of diabetes complications. This result corroborates previous findings [12, 59] and indicates that the participants in this study took responsibility for their choices and respective consequences. Discipline and proactive approaches to self-management are essential to reducing or preventing the development of diabetes complications. Regular reinforcement of education and motivation of patients could provide in-depth information about the disease and foster the will to mitigate its’ clinical course.

Furthermore, our study findings confirm those of other studies that the use of mobile technologies such as smartphone applications [19], insulin pump [60] and continuous glucose monitor [61] could enhance diabetes self-management in patients. Technology interventions have positive impact on diabetes outcomes such as adherence to self-management activities, glycosylated hemoglobin and diabetes self-efficacy [19]. Therefore, health professionals could recommend the use of mobile health technologies to patients who are capable of using them as they benefit from them.

### Barriers to self-management

The lack of enthusiasm towards regular self-management due to the chronic and dynamic nature of diabetes was not entirely unexpected. This phenomenon could be referred to as diabetes distress which is the emotional stress resulting from living with diabetes and the ‘‘burden of relentless management” [62]. High diabetes distress results in sub-optimal diabetes management and compromised quality of life [3, 63]. Diabetes distress is common among patients and impacts on their self-management and health outcomes. Therefore, the importance of providing appropriate regular support to all patients in this regard cannot be overemphasized. Health professionals could ask patients at every consultation about how they are coping with diabetes, encourage them to express particular diabetes related issues causing them distress and offer encouragement and suggestions on ways to deal with it on a daily basis.

For many of the respondents in this study, the need to meet up with job requirements especially frequent travelling, makes adherence to healthy eating difficult. Additionally, work related stress impacts greatly on their blood glucose levels. These findings echo the results of Chao et al. [39]. Recommendations to patients to engage in creative planning and social support are strategies to help address this barrier. Social support from families are essential. Families should be encouraged to attend educational training sessions with patients so as to offer appropriate support which can assist patients to make healthy food choices and decisions regarding their diabetes management [12, 13].

Furthermore, financial burden associated with diabetes could be a hindrance to self-management especially those associated with out-of-pocket expenditure for medical needs. Campbell et al., (2017) observed that the predominant area of management where patients experience financial burdens are medications, diabetes supply and healthy food [37]. People with diabetes require regular self-management and clinical monitoring to prevent the development of complications and foster optimal health outcomes; hence the associated financial demand. Health care providers could inform patients about resources available to them to buffer financial constraints that limit adherence to treatment plans. Such resources may include referring patients to specific social programs or compassionate relief programs to support financial burdens and enable easier access to necessary services.

Differences in patients’ and health care professionals’ (HCP) views of what constitutes the best approach to care was also identified as a barrier to self-management. This may be due to gaps in the way treatment recommendations were communicated to patients. Often times, HCPs’ view of good care are based on adhering to stipulated biomedical care model, structured communication and central decision making [64], whereas patients perceived quality health care is how the scientific knowledge of HCPs’ aligns with their own experiential knowledge and personal preferences [65]. Therefore, patients are always seeking exhaustive information about their diagnosis and treatment [65]. There is responsibility on the part of HCP’s to advice and educate their patients on different treatment options and the reasons they are placed on a particular option and not the other. This patient centered-approach will empower patients and foster their health outcomes.

### Integration of findings and recommendations for future interventions

The survey results show that many patients have limited capacity for healthy coping strategies to identify and manage the impact of diabetes related stress. This finding was confirmed in the interviews where diabetes distress was reported as a major barrier to self-management. Given that stress is a potential contributor to chronic elevated blood glucose levels, it is essential for health care professionals to assist patients with identifying approaches to reducing diabetes distress. Additionally, increased access to healthcare providers through expanded clinic hours could be a means of easing the burden of diabetes diagnosis [41].

The quantitative data also showed that higher educational level was the strongest predictor of better self-management skills in patients and this was affirmed by the highly skilled interviewees who identified the use of technological devices as an enabler to their self-management. This corroborates that higher educational level is a good predictor of eHealth usage [66]. In addition, in accordance with previous literature [67], good overall self-efficacy level observed in the survey might have influenced the positive report on the usefulness of technology in diabetes management. Therefore, given that use of health technologies provides both short and long term health improvement in diabetes patients [68], active usage should be encouraged where necessary especially among patients who are educated and have the ability to engage with them. Furthermore, it is important to device avenues to improve patients’ self-efficacy in their ability to manage the disease as this could increase their likelihood of engaging with technology for their self-management [69].

The interviews revealed that determination to prevent development of complications is one of the major enablers to diabetes-self-management. This might explain the overall high score in skills and self-efficacy observed in the survey. Therefore, we suggest that educators could focus on improving patients’ skills and self-efficacy for diabetes self-management thereby raising patients’ awareness of the negative effect of diabetes. This approach could in turn stimulate the patients’ determination to engage in diabetes self-management and thereby reduce their risk of developing complications.

A unique perspective from the qualitative results revealed that patients and HCPs have divergent views/opinions about what should constitute patient care. It is therefore, imperative that HCPs ensure that patients understand the reasons for the recommended treatments and engage them in shared decision making which is essential for patients’ satisfaction and engagement in self-management practices [70].

Lastly, it has been advocated that people with diabetes should receive self-management education and support in an ongoing and consistent manner [71], but the reality of facilitating face-to-face diabetes education between patients and HCPs on an ongoing basis is low due to limited human and organisational resources. Health behavioural treatments and therapies such as diabetes self-management education/support could be provided to patients on an on-going daily basis outside the clinical setting through the use of ecological momentary interventions such as mobile technologies [72]. Apart from the fact that apps were opined by patients to enable self-management in this study, the World Health Organisation (WHO) also confirmed that the use of mobile technologies (such as apps) can support attainment of health outcomes which could transform health service delivery globally [73]. Considering that Apps are cost effective avenues for providing ongoing delivery of care to patients outside the clinical environment [74], diabetes self-management educational (DSME) messages could be developed and integrated into apps for patients. Such DSME should be targeted at improving patients’ skills and self-efficacy capacity for effective self-management.

### Strengths and limitations

The strength of this work is that it provides a multinational picture of skills and confidence for self-management in people with type 1 or type 2 diabetes. Such an elaborate and international approach to assessing the capacity and confidence levels for self-management is scanty in the literature. In addition, the data identified a number of factors serving as enablers and barriers to diabetes self-management emanated from patients’ perspectives and their lived experiences. Therefore, the results are tenable for providing immense insights into improved strategies for supporting patients in their self-management.

There are some limitations to this study. Firstly, the reliability and validity of the quantitative tool used have not been previously demonstrated at multinational/multicultural levels, therefore, this may limit the interpretation of our findings. Although, in a previous study [32], the construct validity of the scale was tested among type 1 and type 2 diabetic patients who were from different ethnic backgrounds (Asians, Caribbeans, Caucasians etc.), but living in the same regional location. The study reported that the scale was not influenced by ethnicity. Secondly, the small sample size/groups for the survey which mainly comprised of participants from three continents, may limit the generalization of our findings to other settings. Thirdly, the quantitative data were self-reported and therefore susceptible to bias, which may not reflect participants’ actual skills and confidence levels for self-management. Hence, under or over reporting could result in inaccurate identification of common gaps in skills and confidence requiring intervention. Nevertheless, self-report can be made more reliable when questions are asked in a non-judgmental manner as obtained in the SCPI tool used in this study. Lastly, the small number of interview participants is also acknowledged and the interview sessions were brief because additional compensation was not offered to interviewees. Short interview duration was utilised to foster increased participant numbers because long interviews may not be justifiable for participants’ time involvement in the study. Published literature has shown that the anonymity of telephone interview reduces interviewer bias which makes the interview setting more calming and forthcoming, thus fostering a more accurate and truthful data collection [75].

### Conclusion

This study identified common gaps in the skills and self-efficacy of people with type 1 or type 2 diabetes mellitus as well as other perceived enablers of, and barriers to, self-management in this population. Diabetes health care stakeholders may consider strategies for regular educational reinforcement in patients in order to foster healthy coping with diabetes stress, exercise planning to avoid hypoglycemia, interpreting blood glucose patterns and adjusting medications or foods to reach the targeted blood glucose levels. Furthermore, designing of interventions that capitalize on how to improve patients’ desire to reduce the progression of diabetes and the use of relevant technological devices could enhance diabetes self-management. Improved approaches to address diabetes distress, financial burden, discrepancy between patients and their health professionals’ perception of care as well as work and environment related factors are essential to foster improved self-management in patients. Finally, attention should be paid to type of diabetes, level of education and duration of diagnosis when counselling patients on diabetes self-management. Consideration of these areas of educational reinforcement and interventions could enhance self-management in patients and consequently improve their health outcomes.

## Supporting information

S1 Checklist COREQ checklist. (PDF)

S1 Appendix Interview guide. (PDF)

## Abbreviations

SD: Standard deviation
T1D: Type 1 Diabetes mellitus
T2D: Type 2 Diabetes mellitus
