# Supplementary material for: Enablers and barriers to effective diabetes self-management: A multi-national investigation
Source: PLoS One. 2019 Jun 5;14(6):e0217771. doi: 10.1371/journal.pone.0217771 (PMC6550406; doi:10.1371/journal.pone.0217771)
Supplement: S1 Appendix — (PDF) [file pone.0217771.s002.pdf]

Additional File 1: Interview guide

Prompts:

General prompt on key areas of diabetes self-management. This includes adherence to medication intake, regular participation in physical exercise, eating the ideal diet, regular monitoring of blood glucose, problem solving such as correcting hypoglycemia and hyperglycemia, healthy coping with the condition, reducing risk to help prevent or minimize diabetes complications (for example limiting alcohol intake, smoking cessation, regular eye examination, foot care, oral health).

For those who have type 1 diabetes, In addition to the above, other areas of diabetes management was buttressed including with adjusting insulin and diet to avoid hypoglycemia/hyperglycemia, adjusting insulin/exercise to avoid hypoglycemia.

Facilitators

1. Can you identify some strategies you find particularly helpful to facilitate or support you to perform your diabetes self-care activities?
2. Are there things (people or services) that have made it easier for you to manage your diabetes?
3. What are the factors that have contributed to success in your self-management
4. Anything else to add?

Barriers

5. What areas (if any) of your diabetes management do you find difficult?

   Probe: What makes this /these (mention the area(s) stated) difficult?

6. On a day to day basis, is there anything you struggle with or find difficult in your diabetes management?
7. Are there factors that have served as obstacles to managing your diabetes?
8. What do you believe are the barriers to your self-management?
9. Anything else to add?
